# Traditional agricultural management of Kam Sweet Rice (*Oryza sativa* L.) in southeast Guizhou Province, China

**DOI:** 10.1186/s13002-022-00528-6

**Published:** 2022-04-07

**Authors:** Chunhui Liu, Yanjie Wang, Xiaoding Ma, Di Cui, Bing Han, Dayuan Xue, Longzhi Han

**Affiliations:** 1 College of Life and Environmental Sciences, Minzu University of China, Beijing, 100081 People’s Republic of China; 2 Institute of Crop Sciences, Chinese Academy of Agricultural Sciences, Beijing, 100081 People’s Republic of China

**Keywords:** Glutinous rice, Traditional agriculture, Dong ethnic group, Complex ecosystem

## Abstract

**Background:**

The Dong people mainly live in Hunan, Guangxi and Guizhou provinces, China, with a long history of glutinous rice cultivation, among which Kam Sweet Rice (KSR) is a group of rice landraces that has been domesticated for thousands of years by the Dong people. The core distribution area of KSR is Liping, Congjiang and Rongjiang County of southeast, Guizhou Province. Paddy fields, forests, livestock and cottages have formed a special artificial wetland ecosystem in local area, and the Dong people have also formed a set of traditional farming systems of KSR for variety breeding, field management, and soil and water conservation. However, this traditional agricultural management has not been reported at multiple levels based on landraces, species and ecosystems.

**Methods:**

Fieldwork was conducted in ten villages in southeast Guizhou from 2019 to 2021. A total of 229 informants were interviewed from the villages. Semi-structured and key informant interviews were administered to collect ethnoecological data on the characteristics and traditional utilization of KSR, traditional farming systems and agricultural management of the Dong people.

**Results:**

(1): A total of 57 KSR landraces were recorded as used by the Dong people in southeast Guizhou. We analyzed the cultural importance index (CII) of all KSRs. KSR with high CII often has a pleasant taste, special biological characteristics of cold resistance, disease and insect resistance and high utilization in the traditional culture of Dong people. (2) There is a clear division of labor between men and women in the breeding, seed retention, field management and grain storage management of different landraces of KSR in Dong communities. In order to resist natural disasters and insect pests, the cultivation of KSR is usually managed by multi-variety mixed planting. These agricultural management modes are the result of Dong people’s understanding and adaptation to the local natural geographical environment, as well as the experience and wisdom crystallization of Dong people’s long-term practice. (3) The traditional farmland of Dong People is a typical artificial wetland ecosystem that is planted with mixed KSR landraces with rich traditional wisdom. In addition, the economic benefit of the rice–fish–duck symbiotic system was 3.07 times that of hybrid rice alone; therefore, the rice–fish–duck system not only has the function of maintaining soil, water and ecological balance but also improves the income of Dong people.

**Conclusion:**

KSR is a special kind of rice that has been domesticated and cultivated by Dong people for thousands of years. Dong people have also formed traditional agriculture dominated by KSR cultivation. The traditional agricultural management of Dong people provides suitable habitats for flora and fauna with biodiversity protection, and convenient conditions for rational utilization and distribution of water resources were also provided. This traditional management mode is of great significance for environmental protection, climate change response, community resource management, sustainable utilization and agricultural transformation in modern society. Therefore, we call for interdisciplinary research in natural and social sciences, in-depth study of the ecological culture of ethnic areas, and sort out treasures conducive to the development of all mankind.

## Background

Culture can be defined as the product of social development influenced by a specific geographical background and as the result of interactions between the natural environment and social environment [1]. Rich cultural diversity has arisen from the long-term practice of exploiting and utilizing natural resources in manners unique to specific cultural backgrounds [2, 3]. At the same time, as human civilization has progressed, an increasing number of cultural factors are integrated into natural resources [1]. However, while social development has brought economic and social benefits, it has also resulted in diminishing natural resources [4]. As a part of nature, human beings are obligated to use their knowledge and wisdom to improve the environment in addition to having the right to enjoy the gift of nature. People living in ethnic minority areas in China or other indigenous and local communities in the world all have their own unique traditional way of livelihood and thinking, and they have a more direct and closer relationship with the surrounding natural resources [5]. They have rich traditional management experience in natural resources for a long time. This classical traditional management knowledge is the production of life experience summarized by the long-term integration of man and nature [6, 7]. Traditional management research focuses on forest management at the beginning of the twenty-first century [8]. In some traditional communities, forests provide a variety of food and medical resources for the local population [9]. Understanding the traditional knowledge of plant utilization and domestication mastered by communities [10] and supporting the protection, inheritance, access to genetic resources and benefit-sharing of this traditional knowledge are main research hot spots [11]. Adaptive management has been increasingly researched since 2010 [8], and the role of traditional management in maintaining biodiversity is increasingly recognized by policy-makers, conservationists and scientists [12]. Yang et al. [13] studied Hani terraced fields in Yunnan, China, and found that Hani communities effectively resolved the challenges of uneven distributions of water resources through their traditional water resource management system, which represents a significant local response to climate change that promotes sustainable agricultural development. Pradhan et al. [14] showed that the traditional management of sacred forests is compatible with the local culture in Odisha, India, and effectively protects cultural diversity and biodiversity. In recent years, reports have been produced on home garden agroecosystem management [15] and forage resource management [16], and the role of culture in these traditional ecosystem management systems has been extensively studied. In addition, some scholars have conducted comprehensive studies on the role of community calendars in traditional ecosystem management and noted that calendrical systems have been instrumental in guiding the development and management of anthropogenic biomes [17]. In a word, experience in the management of natural resources is of far-reaching significance to the development of modern economic society and the protection and sustainable utilization of natural resources in the future [18].

As a part of natural resources, agricultural genetic resources are deeply influenced by traditional culture and traditional knowledge [19]. Ethnic minorities have cultivated a large number of crop variety resources in thousands of years in their agricultural production practice, greatly enriching agricultural biodiversity [20]. At the same time, they have created rich traditional culture, innovation and practice in their long-term production and life [5, 6], most of which have important social, economic, cultural and ecological value [21]. Landraces are an important part of agricultural genetic resources with distinctive characteristics [22]; without formal genetic improvement, they often have relatively good resistance to stress, disease and pests [23, 24] and adapt to traditional cultivation methods and culture. The formation of landraces is closely related to the cultivation methods, traditional culture and human activities of cultivators [25]. Yamanaka et al. [26] observed that people living in the mountainous areas of the Indochina Peninsula in Southeast Asia showed a preference for glutinous rice, which is part of their traditional culture, and their cultivation process bred multiple glutinous rice landraces. The provinces located in southwest China, such as Guangxi, Yunnan and Guizhou, where large numbers of ethnic minorities live, are rich in rice landraces [27], especially in Guizhou. Ethnic minorities bred a variety of rice landraces resources through their own unique traditional culture and management methods [28].

In an era of global climate shift, where modern varieties have shown low adaptive potential to changing environmental conditions [29], thus traditional agricultural management of landraces have regained the increased attention worldwide as the sustainable agriculture systems [30]. Abate et al*.* [31] pointed out that farmers' traditional agricultural management of landraces is a built-in process in the overall crop production system rather than a separate well-defined activity, it is determined by the social form, traditional culture and economic environment of farmers, and maintains the diversity of landraces through their own cognition, knowledge, resource and practice [32]. Traditional agricultural management practices of landraces have positive effects on genetic diversity [33], seed network systems [34], pest control and management, food nutrition and health [35] and so on. High productivity, biodiversity conservation, low energy inputs and climate change mitigation are some of the salient features of the traditional agriculture systems [36]. Farmers’ age and gender, kinship structure, income level, education level and language are the main factors affecting traditional agricultural management [32], while traditional practices like agroforestry, intercropping, crop rotation, cover cropping, traditional organic composting and integrated crop-animal farming are the main way of traditional agricultural management [30].

The Dong people are one of China’s ethnic minorities, and their main residential area is the border of Guizhou, Hunan and Guangxi provinces. Their main traditional livelihood is agriculture, which is one of the ancient rice-farming nations of humankind [37, 38]. They produce mainly glutinous rice and domesticate their nationality’s unique and high-quality variety called—Kam Sweet Rice (KSR), which has the characteristics of a strong fragrance, waxiness, hard threshing and resistance to stress. It is cultivated and shaped by Dong people in thousands of years of production and life practice and cultural customs [39]. With the development of breeding technology, modern breeds are constantly replacing landraces, but KSR has been preserved and cultivated continuously in the Dong community, because KSR is closely related to the traditional food culture, festival culture, religious sacrifice and other aspects of the local Dong people [40]. Additionally, it is one of the representative materials of Dong traditional culture [41]. Dong people not only have made KSR a staple food, as an auxiliary material for meat curing and glutinous rice wine, but also use it in various important festivals and sacrificial activities. No traditional festivals would occur without KSR [42, 43]. KSR can also be used for the exchange of goods, acting as money [40]. KSR has become the material basis of Dong people's lives and gives birth to their unique ethnic customs, lifestyles and cultural customs. Since 2008, the KSR has attracted scientific interest and was firstly reported in *Science* [44]. KSR was selected as a national geographical indication product of agricultural products. The rice–fish–duck ecosystem in southeast Guizhou was listed in the first batch of globally important agricultural cultural heritages, and they have been elected into the Globally Important Agricultural Heritage Systems (2011) and the UNESCO World Heritage List.

Currently, there are few systematic studies on KSR, some of which focus mainly on variety collection and naming and the relationship between KSR and Dong traditional culture [39, 45]. Lei et al*.* [39] identified a total of 91 KSR landraces according to the traditional folk classification method and believed that they had high genetic diversity. Zhou et al*.* [45] found that KSR has a very ancient historical origin with Dong people, and the traditional culture of Dong people is closely related to the continuous planting and grain quality formation of KSR. However, there are few systematic studies on the traditional agricultural management of KSR by combining qualitative and quantitative methods. What are the cultivation and utilization methods, storage management and field management methods of KSR in the Dong community? What are the scientific implications of the rice–fish–duck symbiotic ecosystem of KSR paddy? It is not clear. Therefore, this study will sort the traditional agricultural management knowledge and experience of KSR from three levels of landraces, species and ecosystems, revealing the status and role of KSR in traditional ecological agriculture under the traditional cultural background of the Dong people, and the important ecological significance and scientific value of the traditional agricultural management mode of the Dong people to the protection of natural resources, in order to provide scientific and technological support for the protection of KSR resources and the sustainable development of Dong traditional agricultural management.

## Methods

### Study area

**Fig. 1:**
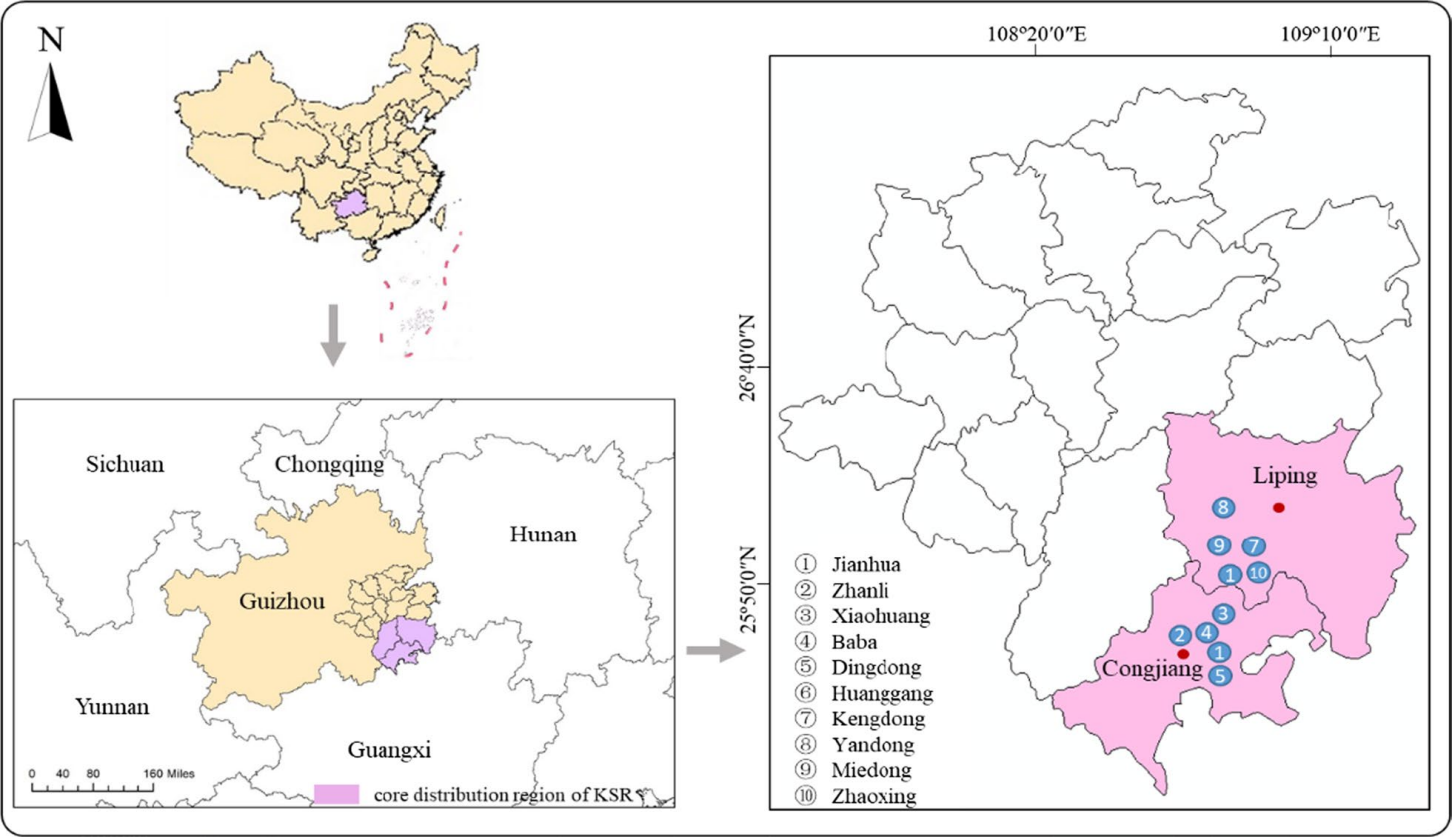
Location of the study areas

Ethnoecological studies were conducted in ten Dong villages in southeast Guizhou (Fig. 1). Southeast Guizhou is located at 107° 17′ 20″–109° 35′ 24″ east longitude and 25° 19′ 20″–27° 31′ 40″ north latitude. The total population of the Dong nationality accounts for 30.5%. Southeast Guizhou is the main settlement area of the Dong nationality in China. It has a subtropical humid monsoon climate, with an annual average temperature of 14.7–18.5 °C and annual precipitation of 1032.5–1456.8 mm. There is no severe cold in winter or severe heat in summer. There are four distinct seasons, abundant rain and an obvious three-dimensional climate. The Dong people speak their own language, which does not have a traditional writing system. After 1958, a set of writing characters of the Dong language was created with the help of the Chinese government and linguists. The population of the Dong ethnic group in China is 3.496 million according to the China Statistical Yearbook 2021 [46], among which southeast Guizhou is the Dong ethnic group’s largest residential area in China. The ten villages are under the jurisdiction of Congjiang and Liping County. Congjiang County has the largest planting area of KSR in China [40] (39.9% is the Dong population), and Liping County has the largest Dong population in China. (69.9% is the Dong population.)

Glutinous rice cultivation has a long history in Congjiang and Liping. The local KSR and Dong traditional culture infiltrate each other to form a unique farming and glutinous rice culture, which is reflected in the production and life, cultural customs, religious sacrifice and other aspects of Dong people. They are the main production area of KSR and an important birthplace of glutinous rice culture.

We selected these ten Dong villages as the case study sites according to the selection principle of ethnobotanical survey sites [47], and the details of these villages are shown in Table 1. The ethnic composition of these villages is mainly Dong people, and they have a long history of settlement; each village has more than 100 households. The surrounding ecological environment and traditional culture are well preserved and are less affected by the outside world. Additionally, the elevation gradient of these villages ranges from 240 to 800 m with a large span, which better reflects the three-dimensional climate characteristics of southeastern Guizhou. The above information shows that these villages are ideal areas to study the knowledge of KSR traditional ecological agriculture of the Dong people.

**Table 1:** Information of ten Dong villages in southwest Guizhou

County	Township	Village	No. of families	Population	Altitude (m)	Nationality	Area of KSR paddy field (ha)	Proportion^a^ (%)
Congjiang	Gaozeng	Jianhua	165	800	800	100% Dong	33.3	83
	Gaozeng	Zhanli	186	829	380	100% Dong	40.0	80
	Gaozeng	Xiaohuang	820	3700	630	100% Dong	40.0	50
	Gaozeng	Baba	280	1200	600	100% Dong	33.3	65
	Xishan	Dingdong	500	2070	240	100% Dong	62.0	80
Liping	Shuangjiang	Huanggang	360	1629	780	95% Dong	66.7	67
	Shuangjiang	Kengdong	381	1585	330	100% Dong	60.0	50
	Yandong	Yandong	980	4238	757	96% Dong	53.3	35
	Shuangjiang	Miedong	302	1400	434	100% Dong	21.7	35
	Zhaoxing	Zhaoxing	1100	6100	610	100% Dong	Scattered planting	–

^a^Proportion of KSR planting area to total paddy area

### Material collection

61 KSR samples were collected in ten villages from 2019 to 2021, among which 4 KSR were shared by two villages. The sample collection method refers to the *Technical Regulations for Crop Germplasm Resources Collection* compiled by Zheng et al*.* [48]. All the collected samples are deposited at the National Gene Bank.

### Data collection

**Fig. 2:**
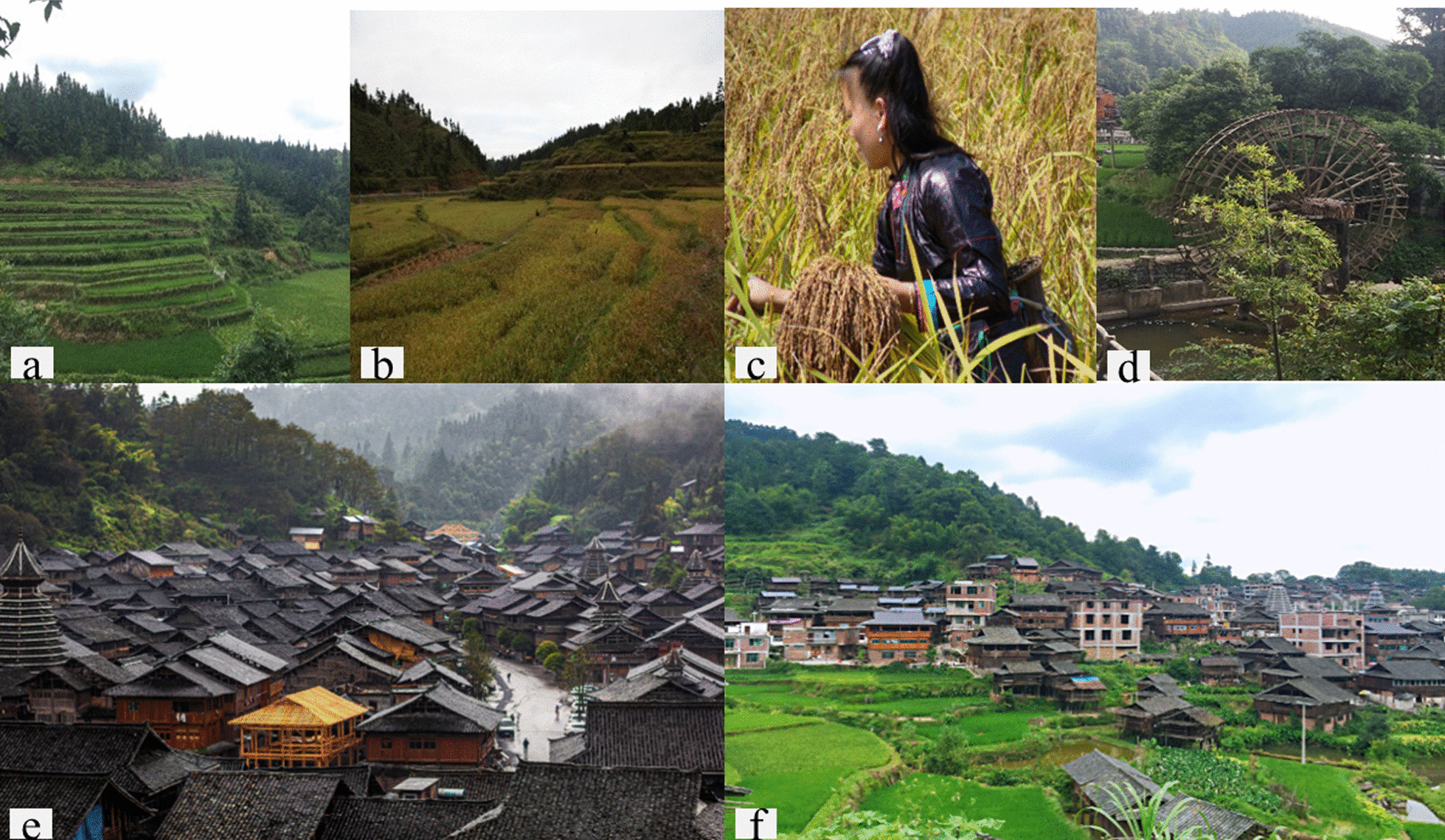
**a**–**c** KSR paddy fields at different stages; **d** use a traditional water wheel for irrigation. **e** Terraces hidden deep in forest in Huanggang; **f** terraces surround the cottages in Zhaoxing

Ethnoecological surveys were conducted in July 2019, August 2020 and October 2021, which were the tiller stage, heading stage and mature period of KSR, respectively (Fig. 2a–c). Through the snowball technique, a total of 229 respondents (128 males and 101 females) were interviewed in ten Dong villages, including key informant and semi-structured interviews. For the purpose of this study, we selected farmers engaged in KSR cultivation as informants. Survey sites include but are not limited to farmers' homes, farmland, fish ponds, streets and workshops. A total of 36 key informant interviews were interviewed, including local experts, village cadres, clan elders (village elders, headmen, etc.) and inheritors of intangible cultural heritage. The semi-structured interviews involved open-ended questions and conversations with informants in the above scenes.

The major questions of our surveys are as follows:

1. How many landraces of KSR do you plant now? What are the names?
2. What are the characteristics of these landraces? Does it taste better? Or is it cold-resistant, disease-resistant and of high economic value?
3. Do you prefer KSR or hybrid rice? Why is that?
4. How many landraces of KSR did you have in the past? Why are they increasing or decreasing now?
5. Which of these landraces of KSR are grown now for staple food?
6. Which landraces of KSR are often used in traditional festivals and religious sacrifices?
7. Is your family’s main source of income the sale of KSR?
8. Can you tell us how the KSR terraces are built? What is the function?
9. Do you raise fish and ducks in your KSR field? What do you think of this approach?
10. What other interesting things can you share with us?

Interviews were conducted in the Dong language or simple Mandarin with the assistance of local Dong translators. All interview procedures involved in this study were in accordance with the International Society of Ethnobiology Code of Ethics, including procuring prior informed consent before interviews [49]. The demographic characteristics (age, educational status and occupation) were identified and recorded in all face-to-face interviews (Table 2).

**Table 2:** Demographic details of interviewed informants

Category	Subcategory	No	Proportion (%)
Gender	Male	128	55.9
	Female	101	44.1
Age	20–40	54	23.6
	40–60	101	44.1
	60–80	68	29.7
	80 and older	6	2.6
Education status	Illiterate	114	49.8
	Primary	71	31.0
	Secondary	23	10.0
	Higher	21	9.2
Occupation	Farmer	162	70.7
	Migrant workers	41	17.9
	Local officials	26	11.4

In addition to interviews, we conducted participatory observations in the study site communities. Specifically, we devoted ourselves to the life and production of local ethnic minorities, focusing on the layout of the traditional agricultural ecosystem of the Dong people, the traditional management of farming and the daily production and collective activities of the Dong people, to deepen the understanding of the connotation of traditional knowledge and grasp traditional knowledge more accurately.

### Data analysis

#### CII analysis

**Fig. 3:**
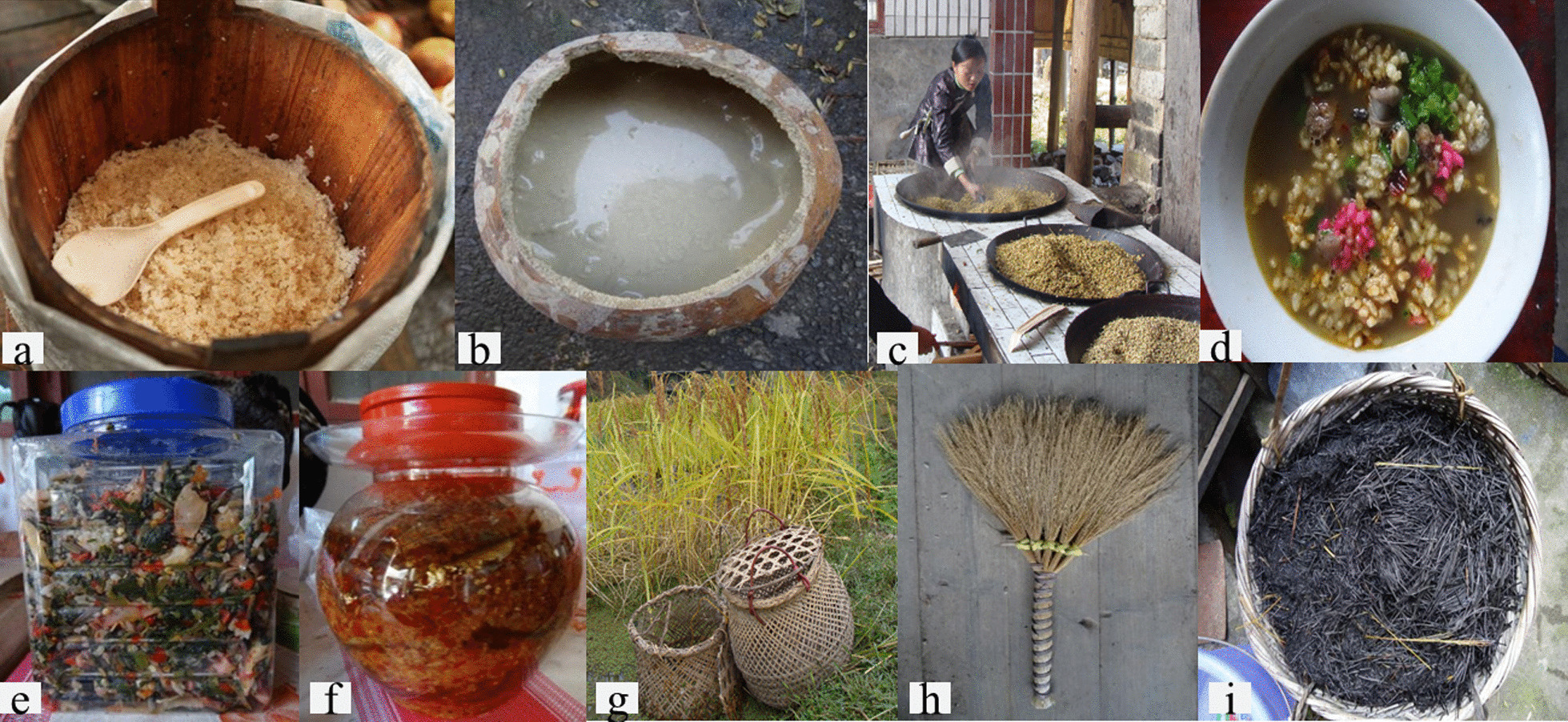
Traditional knowledge of KSR: **a** KSR as a staple food; **b** a sour soup made of glutinous rice; **c**, **d** fried rice and oil tea made from KSR; **e**, **f** salted vegetables and fish; and **g**–**i** fish cages, brooms and grass ash made from stalks

Based on the interview results, we divided the traditional knowledge of KSR in seven ways (Fig. 3): staple food (glutinous rice), snacks (drinks, rice dumpling, fried rice, etc.), auxiliary materials (salted fish, vegetables, etc.), festival celebration, religious sacrifice, medicinal plant, straw as dye and weaving materials. The cultural importance indices (CIIs) [50] of each KSR variety were calculated to evaluate the integrated value of the KSR. It is defined as the sum of the percentage of respondents who mentioned each use of a useful plant. This additive index accounts for not only the spread of the use (number of informants) for each species but also the diversity of its uses.

The formula for CII is:

$$CII_{s} = \mathop \sum \limits_{{u = u_{1} }}^{{u_{NC} }} \mathop \sum \limits_{{i = i_{1} }}^{{i_{N} }} \frac{{UR_{ui} }}{N}$$

where s is a variety of KSR, named s, u is a traditional utilization, named u, N is the total number of informants, and *UR*_*ui*_ is mentioned by informant i in traditional utilization u.

## Results

### Traditional agriculture based on KSR cultivation

#### Variety diversity of KSR

A total of 61 KSR landraces were collected in ten Dong villages (Table 3), among which 14 landraces were cultivated on a large scale and widely used, 4 landraces were shared by two villages, and the remaining 57 landraces were different. The main KSR landraces planted by Dong people are also different in every Dong village due to the differences in geographical environment, dietary culture and other traditional cultures. In Huanggang Village, for example, “Liezhuhe” is the most widely grown variety in the area due to the dense jungle, short sunshine duration and high elevation of the paddy fields. This variety has a short awn, small grain shape and strong adaptability to cold conditions. “Baixianghe” is the main local variety in Yandong Village, which is suitable for planting at low altitude, has a strong fragrance and is suitable for the traditional cultural needs of the local Dong people. In addition, Dong people take mainly KSR as their daily staple food in some villages, such as Huang Gang, Jianhua and Zhanli; therefore, the planting area is large. In other villages, KSR appears in only Dong traditional cultural festivals and religious sacrifices; thus, the planting area is small.

**Table 3:** Inventory of KSR species in southeast Guizhou and their CII

Village	No	Local name	CII	Cultural values of KSR in this village
Jianhua	1	120-day he	1.18	These landraces of KSR are kept by farmers for decades or hundreds of years The most important festivals in Jianhua are the Dragon Boat Festival, the Zongba Festival and Xinmi Festival Every family will steam glutinous rice and drink glutinous rice wine to celebrate the festival. They will also make glutinous rice pickled fish and sour soup to celebrate the festival The small amount of hybrid rice grown by the Dong people in Jianhua are used only to feed livestock or entertain guests who do not like KSR Local herbalists also add KSR to some herbs to enhance their efficacy
	2	130-day he	0.45	
	3	Black he	1.77	
	4	Goucengao	1.68	
	5	Gouyangdang	4.05	
	6	Goutunrong	0.91	
	7	Gouliejiu	1.45	
	8	Gouliezhu	1.86	
	9	Gouliedainian	1.86	
	10	Gongmuhe	1.09	
	11	Goucengaoka	5.05	
	12	Gouhuanggang	1.00	
Zhanli	13	Wangni	1.22	Zhanli village is famous in China for having only one boy and one girl in every family, and its natural population growth rate remains close to zero The most important festival in Zhanli is the first day of February and the first day of August, when every family will exchange different KSR as gifts Dong people often weave straw ropes and sandals from KSR stalks and burn them into grass ash for dyeing cloth
	14	Gouliezhu	0.89	
	15	Zhanliheinuo	1.22	
	16	Yangdanghe	1.00	
	17	Yansanse	0.94	
	18	Gouhada	0.56	
	19	Dabaohe	0.94	
	20	Gonggu	0.56	
	21	Zhanlinuohe	2.39	
	22	Danglao	1.78	
Xiaohuang	23	Red he-1	2.84	The mid-autumn day is the most solemn festival of the year in Xiaohuang, every family steamed glutinous rice, drank glutinous rice wine and so on Dong people also use KSR to extract maltose and make candy in daily life
	24	Yangwenna	0.52	
	25	Dabaohe	1.04	
	26	Gouhagongniu	1.48	
	27	Goubadang	0.52	
Baba	28	Liezhuhe	3.93	Baba village is rich in folk traditional culture, with various types of performances and folk activities The Xinmi Festival and Spring Festival are more important festivals in Baba, and the villagers will make different foods with KSR to celebrate Baba village has developed into an ethnic tourism village and has exchanges with the outside world; thus, KSR landraces have been exchanged with other villages
	29	Babagonghe	0.79	
	30	Danianhe	1.21	
	31	Jiuyuejiu	1.00	
	32	Gougong	1.14	
	33	Gouhadang	1.21	
Dingdong	34	Gouyongmi	1.48	Dingdong village is a relatively wealthy Dong village Selling KSR is an important livelihood for farmers. The better landraces are sold at a price of 4.76$/kg in the market The grandest festival in Dingdong village is “Adult Day” on July 14, which is mainly held for young men and women aged 16–18, every family eats KSR
	35	Gouyongwai	0.92	
	36	Dingdongheihe	1.72	
	37	Goudainian	0.80	
	38	Goudang	0.68	
	39	Xianggu	0.72	
Huanggang	40	Red he	2.12	Every family grows KSR and raises fish, ducks in Huanggang village, and is famous for its rice–fish–duck ecosystem Dong people in Huanggang are particularly fond of eating KSR, which is indispensable for all meals and festivals The grandest festival in Huanggang village is “Hantian Day” on June 15. Dong people would invite friends from neighboring villages to participate in the festival, eating glutinous rice and drinking glutinous rice wine Villagers in Huanggang do not eat hybrid rice and use it only for tourists
	41	Liezhuhe	4.79	
	42	Old-Liezhuhe	0.39	
	43	60-day he	1.36	
	44	70-day he	0.61	
	45	Jindongnuo	0.33	
	46	Huanggangyangnong	0.55	
Kengdong	47	Heimanghe	0.95	KSR is mainly used for market sales, festival celebrations or as gifts in Kengdong The most important festival in Kengdong is the “Shuaijiao Festival” on February 15. Villagers fry or steam KRS, which is not completely ripe, as a snack
	48	Niumaohe	2.53	
	49	Tonghe	0.84	
	50	Dongronghe	2.89	
	51	Gonggenghe	2.79	
Yandong	52	Wuminghe	2.52	KSR is mainly used to make snacks such as glutinous rice oil tea in Yandong village, which Dong people drink in the morning and afternoon every day The most important festival in Yandong is the Black Rice Festival on April 8, when the KSR is dyed black with leaves of *Vaccinium bracteatum Thunb*. and steamed
	53	Baixianghe	4.29	
	54	Huangshanxue	2.00	
Miedong	55	Wuminghe	2.06	The KSR in Miedong village is mainly used in festivals, such as Zongba Festival on June 6 and July 15
	56	Gouzhaige	3.75	
	57	Ougen	1.19	
Zhaoxing	58	Black nuo	2.61	Zhaoxing village is the biggest and oldest Dong village in China, The village is a wonderland for Dong festivals, songs and dancing. The most famous event is the Grand Song, a unique polyphonic folk music tradition that has been passed from generation to generation for over 2,500 years, the KSR in Zhaoxing is used mainly in these activities
	59	Hongmangbainuo	2.86	
	60	Changmangdanuo	0.81	
	61	Wumangdanuo	0.44	

#### The CII value of KSR in southeast Guizhou

Although the number of landraces of KSR planted was reduced compared with our team's previous research [40], each household will continue to plant four–six different landraces for Dong people in most villages. Therefore, the cultural importance index (CII) of all KSRs was calculated using quantitative methods to determine their importance. The CIIs of 61 species are listed in Table 3.

KSR with a high CII often have adapt well to the environment. These dominant landraces have a pleasant taste and strong fragrance and are staple foods of Dong people, such as “Goucengengka” in Jianhua village, “Liezhuhe” in Huanggang village and “Niumaohe” in Kengdong village. These KSR landraces are not only used in the traditional diet of Dong people, but also can meet the local cultural and play an intrinsic role in the life of Dong people, they are fully utilized in food culture, festival celebrations and religious beliefs. For the Dong people, KSR is the most precious rice and is given to others as a gift for a newborn, when young people get married, and for other important traditional festivals. In addition, KSR is necessary in some sacrificial ceremonies of the Dong people. For these rites, KSR cannot be replaced by other rice or hybrid rice, due to the specific requirements of the sacrificial rites to use KSR. In contrast, some KSR landraces have a high CII values at small planting scales, but their traditional utilization value is also fully played in the specific festivals, culture and religion of Dong people. Only this unique variety can embody its cultural value in the eyes of Dong people. “Dongronghe” of Kengdong village, for example, is neither the staple food of Dong people nor the dominant variety of the village. However, due to its special seed coat color (red) and good taste, it is fully used in the traditional festivals of Dong people. Dong people will make colored glutinous rice, traditional Chinese rice-pudding and so on with “Dongronghe” as raw materials during festivals. Additionally, they believe “Dongronghe” is the most precious. We can screen out variety with high values using the CII, for which on-farm conservation can be enhanced and public recognition of the cultural value of these species can be increased.

### Breeding, storage management and field management of KSR

#### Variety breeding and field management based on male and female division of labor

Dong people engage in different farm work according to different seasons and agricultural terms, and men and women have their own division of labor, forming a set of conventional breeding and field management mode. The second month to the fourth month of the lunar calendar belongs to the stage of preparing for cultivation and raising seedlings. The male labor in the field is mainly to rake the field and build irrigation canals. Women are mainly responsible for clearing weeds near the field, collecting farm manure, selecting seeds and raising seedlings. In terms of variety selection, a family will generally choose 4–6 landraces of KSR. Women mainly choose suitable landraces according to different maturity stages of KSR, adaptability to field soil and resistance to diseases and insects. If a family chooses a slightly larger variety of KSR, they do not plant them all in 1 year, but rotate them over many years. Because different KSR landraces have different requirements for paddy fields, changing the varieties planted in paddy fields every year is beneficial to the balanced utilization of soil nutrients and the control of disease, insect, and also can effectively improve the physical and chemical properties of soil and regulate soil fertility. After the transplant rice seedlings, the Dong people regularly take care of the seedlings, weeding and topdressing, which are usually done by men, Dong women will put the fish and ducks into the paddy fields, forming a rice–fish–duck symbiotic ecosystem when the seedlings grow to a certain stage. The ninth lunar month is the season to harvest KSR, and women will cooperate with men to release water and catch fish to harvest rice. Dong people attach great importance to seed selection and seed retention of KSR. Dong women usually choose the rice ears with full grain and no disease in the field in the mature stage and reserve them as the next year's rice seeds for sowing.

Therefore, male and female labor division is a cooperative and complementary relationship in the field management of KSR, and they formed a set of traditional agricultural management knowledge related to KSR. Males are very familiar with the construction, irrigation of KSR terraces, while females are more familiar with the breeding, growth habits of landraces and traditional use of KSR in life. For example, some black glutinous landraces, the rice stalks are often used by women to make dyes for dyeing cloth, the roots and grains of Huangshanxue are often used as medicinal resources to treat asthma and other diseases. Because of the different division of labor between males and females in traditional agricultural management of KSR, cultural values of KSR are different for males and females. Males paid more attention to the field adaptability and yield of KSR, while females paid more attention to the way of using KSR in traditional culture. These traditional management systems with different emphasis due to gender differences are the important social foundation for the long-term inheritance of KSR (Table 4).

**Table 4:** The division of labor between men and women in traditional management of KSR

Time	Stage	Division of labor	Worker
From Feb to Apr of the lunar calendar	Preparation stage	Rake the field and build irrigation canals	Males
		Clearing weeds, collecting farm manure, selecting seeds and raising seedlings	Females
From May to Aug of the lunar calendar	Seedling stage	Transplant rice seedlings	Males and females
		Take care of the seedlings, weeding and topdressing	Males
		Put the fish and ducks into the paddy fields	Females
Sept of the lunar calendar	Maturation stage	Release water, catch fish and ducks, harvest rice	Males and females
		Seed selection and seed retention of KSR	Females

#### Traditional agronomic management of multi-variety mixed planting

Dong villagers have formed the traditional agronomy of multi-species mixed planting to resist natural disasters and insect pests, which is profoundly ecologically wise. They skillfully use the different biological properties of different landraces of KSR to avoid natural risks and form the unique ecological ethics of the Dong people. On the one hand, the mixed planting pattern creates conditions for cross-pollination between different varieties; thus, other varieties naturally emerge, and Dong villagers will consciously retain such varieties for trial planting. If they can adapt to the local environment, they will expand the planting. After generations of cultivation, they can select and breed new glutinous rice varieties. On the other hand, different varieties mature at different times, and harvesting seeds at different times can avoid the impact of labor shortages to ensure the output and harvest of rice seeds. Generally speaking, each Dong family chooses three or four different landraces of KSR and plants them in the same paddy field. In Huanggang village, for example, Dong villagers will choose Liezhuhe, Jindongnuo and 70-day-he mixed planting; among them, Liezhuhe has wide adaptability and high yield, to ensure enough rice for life. The mature stage of Jindongnuo is late, so it can be reaped after the harvest of Liezhuhe, avoiding the shortage of family labor force. 70-day-he has a strong drought resistance, to avoid no kernels or seeds are gathered caused by extreme dry weather.

#### Traditional storage and management techniques of KSR

**Fig. 4:**
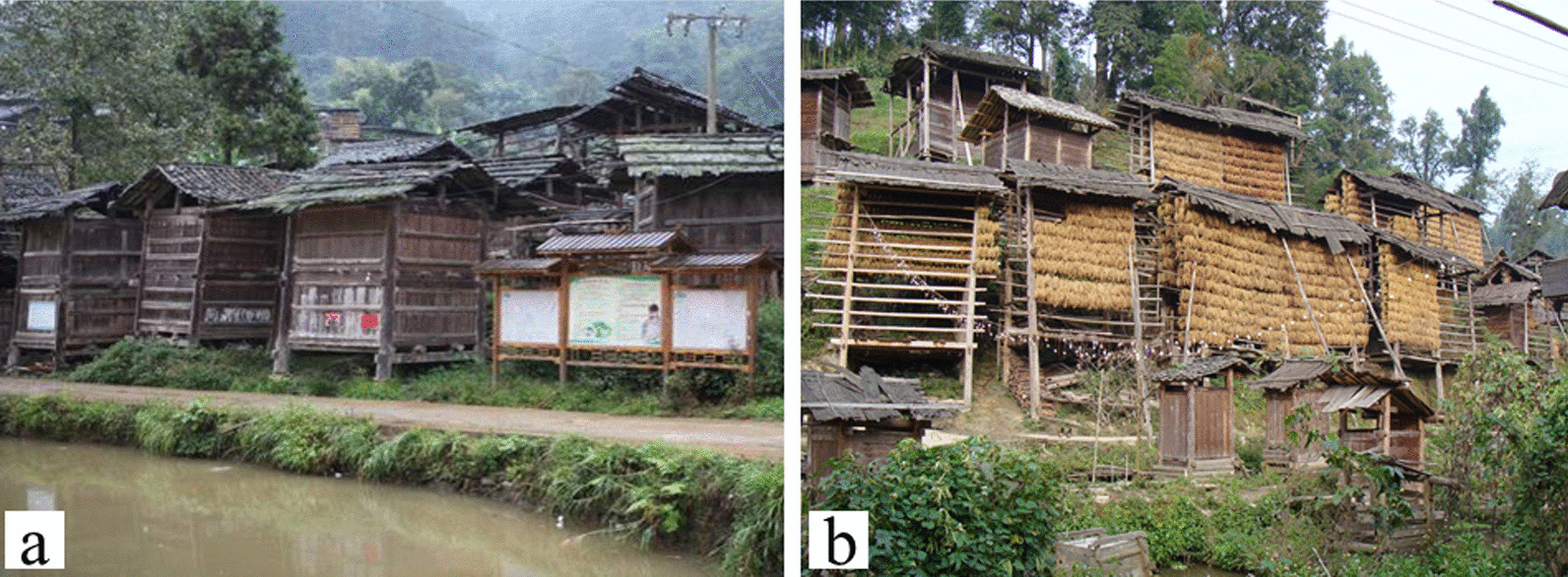
Granary (**a**) and heliang (**b**) were built in a row along the river in Dong village

Threshing is one of the most important part in the harvest process of crops, but it is very difficult to threshing of KSR under natural conditions, the Dong people need to manually harvest with the original picking tool—“half-moon pliers”—and threshing is only carried out when eating. Dong people use half-moon pliers to harvest the ear of rice one by one and tie them into a bunch, then carry them home with a shoulder pole and dry them on the grain rack, which is commonly known as “heliang” in Dong areas, that is a beautiful cultural landscape in Dong villages every October. The seeds are thoroughly dried after 1 month and then stored in a granary. Granary and drying grain rack (“heliang”) are often away from the house (Fig. 4a, b), and “heliang” was built in a row along the river to facilitate drying and ventilation, while avoiding fire, “granary” is not in the house but is built on the pond, convenient for fire protection and convenient for rat. The germplasm resources of KSR are continuously preserved year by year in this way.

Dong people have strict requirements on stored grain, such as removing impurities as much as possible, strictly controlling moisture and strengthening ventilation to avoid mildew of grain. It can be seen that the traditional rice storage technology is closely related to the local Dong people's life style, which is the result of Dong people's understanding and adaptation to the local natural geographical environment, as well as the experience and wisdom crystallization of long-term practice of Dong people, and plays a very important role in maintaining the survival of Dong people.

#### The conventional tillage system of water and soil conservation

**Fig. 5:**
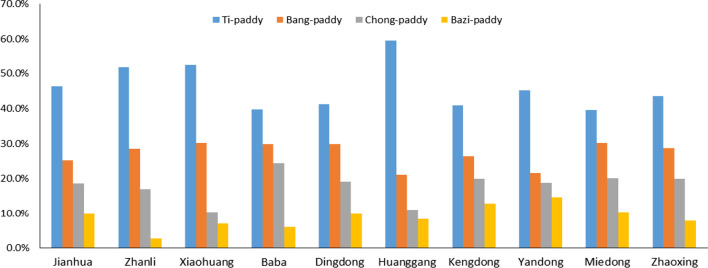
The area proportion of four different paddy fields in ten villages. Terrace fields: the bench section field constructed along a contour line on a hillside; Bang fields: the field on top of a hill; Bazi fields: the field on a partial plain; Chong fields: flat fields between mountains

The paddy fields of Dong people are divided into mainly terrace fields (the bench section field constructed along a contour line on a hillside), Bang fields (the field on top of a hill), Bazi fields (the field on a partial plain) and Chong fields (flat field between mountains) according to Chen's research [51]. The proportion of paddy fields of ten villages with different types is shown in Fig. 5. The KSR fields of the Dong people are mainly terraced fields (“Yav Janc” in the Dong language and “Titian” in Chinese). Dong people build terraced fields into a typical constructed wetland ecosystem using their wisdom of traditional farming techniques. Most of these terraced fields are built on the same level as the hills surrounding the cottages (Fig. 2e). There are also some villages, such as Huanggang, that cannot be connected with the paddy fields due to the high altitude and complicated terrain; hence, the Dong people must build terraced fields in the forests around the village (Fig. 2f). Therefore, the paddy field ecological landscape of Huanggang village is completely different from that of other Dong villages; you will see only the forest but not the field when entering the village.

The Dong people build terraced fields along hillsides to form a three-dimensional structure. Natural spring water is then diverted to the terraces; for the terraced fields along the river, the water is poured into the fields by a traditional water wheel (Fig. 2d). The KSR terraces of the Dong people are a unique natural water cycle ecosystem, and they collect rainwater for terraces all year round. The natural differential pressure is used to supply the water needed by the terraced fields in the rainy season when rainfall is abundant, and the water is irrigated from high to low. Finally, the excess water is discharged into the river from the bottom of the slope. It also replenishes groundwater through osmosis, and the water in the paddy fields naturally evaporates into clouds and rain in the case of the drought season, which helps in regulating the climate. This unique regional microclimate environment not only avoids the greenhouse effect but is also conducive to water and soil conservation. The beautiful ecological environment in the Dong area is formed and preserved in this way. In ten villages we surveyed, most of the terraced fields had thickened and raised ridges, which villagers said were designed to maximize rainwater storage. The KSR planted in the terraces is generally the variety carefully bred and domesticated by the Dong people, and the plant height can reach 1.5–1.8 m; therefore, they are not afraid of flooding and easily store deep water. These KSRs have good resistance to diseases and insects and seldom use pesticides and chemical fertilizers, which also play a positive role in promoting the protection of the local ecological environment. In addition, the growing period of KSR is longer than that of hybrid rice (usually about 160–180 days); thus, terraced fields can be filled with water year round, forming a natural miniature reservoir. The good ecological environment and relatively fewer flood and drought disasters in the Dong nationality area are closely related to this conventional tillage system.

#### Rice–fish–duck symbiotic farmland ecosystem

**Fig. 6:**
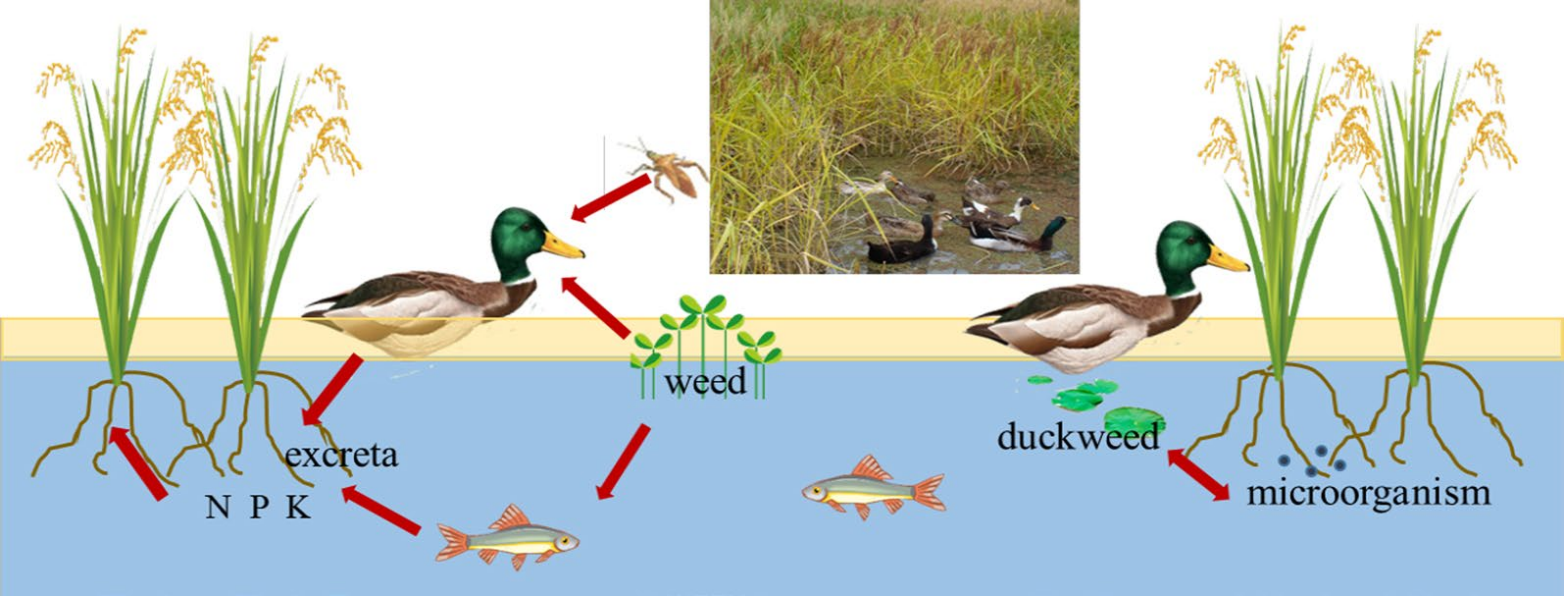
Schematic diagram of the rice–fish–duck symbiotic system

Dong farmers have traditionally raised fish and ducks in KSR fields since ancient times (Fig. 6). KSR landraces in this rechecked ecosystem of the Dong community have the following common characteristics: (1) The variety of KSR is abundant to meet different soil and climate conditions to ensure sustainable farmland ecosystems; (2) these landraces should be tall, not prone to lodging and resistant to flooding, which is not only conducive to water storage in the terraces but also conducive to the free movement of ducks and the growth of fish; (3) the KSR landraces are tolerant of the cold because most of the paddy fields are located in deep mountains and forests where the sunshine hours are few; and (4) these KSR landraces have good resistance to disease and insect pests and do not use pesticides and chemical fertilizers during the growing period to ensure maximum economic benefits and ecosystem security.

Dong villagers actively control the entry time of rice, fish and duck by taking advantage of the time difference in the growing season of biological species to realize the sustainability of the KSR farmland ecosystem. When transplanting rice seedlings, the fish fry is put into the paddy field. The paddy field is generally dominated by carp (*Cyprinus Carpio*), while the fish pond is mostly grass carp (*Ctenopharyngodon idella*). Duck seedlings are put into paddy fields when the fish grow to 4–5 cm, and most of them are local ducks domesticated by Dong people who are small in size and have a high rate of meat and egg production. Fish and ducks not only remove pests and weeds from rice fields and increase oxygen levels in the water, but their excrement is also a good fertilizer for rice growth. Thus, terrace fields, KSR, fish and ducks form a complete wetland ecosystem together. In Dong villages, it is called “Daeml Yav” and “Oux Naemx” [51]. “Daeml Yav” means fish and rice paddy, and “Oux Naemx” means wealth, so this kind of agroecological system is very precious in the eyes of Dong people, which contributes to the rich agricultural biodiversity and the sustainability of the ecosystem. We often see villagers who leave for work always take poultry and livestock out at the same time in our field study. This is the necessary operation of breeding check. There is another plant in this system called the duckweed, and Dong people have unique survival wisdom that they never clean the duckweed in the paddy fields. The microorganisms and duckweed in the traditional paddy field ecosystem could interact and promote each other's growth, maintaining a benign paddy field ecosystem [52].

Although the yield of KSR is not as high as that of hybrid rice, the economic value of the rice–fish–duck symbiotic ecosystem is higher than that of KSR or hybrid rice alone. Referring to previous studies [53], the economic value of paddy fields per hectare was calculated according to data provided by Wu Yusheng, who is a village cadre in Huanggang. The prices are based on 2021 prices ($1 USD = ¥6.3) The results showed that the rice–fish–duck symbiotic farmland achieved the economic benefits of minimum input and maximum income (Table 5), and the economic benefit of the rice–fish–duck symbiotic system was 3.07 times that of hybrid rice alone.

**Table 5:** Economic benefits of rice–fish–duck symbiotic farmland and hybrid rice farmland in the Dong nationality

Item	Category	KSR–fish–duck farmland	Hybrid rice–fish–duck farmland	KSR farmland	Hybrid rice farmland
Income	Rice	16.67 kg × 2.20$/kg = 36.67$	30.00 kg × 0.73$/kg = 21.90$	16.67 kg × 2.20$/kg = 36.67$	30.00 kg × 0.73$/kg = 21.90$
	Fish	1.53 kg × 7.90$/kg = 12.11$	0.90 kg × 4.70$/kg = 4.39$	–	–
	Straw	23.33 × 0.16$/bundle = 3.73$	–	–	–
Expenditure	Seeds	0.07 kg × 6.30$/kg = 0.42$	0.10 kg × 12.6$/kg = 1.26$	0.07 kg × 6.30$/kg = 0.42$	0.10 kg × 12.6$/kg = 1.26$
	Pesticides and fertilizers	–	1.83$	–	3.39$
	Cost of harvesting	3.11$	1.05$	3.11$	1.05$
Total	–	49.82$	22.14$	33.14$	16.20$

The calculation method refers to the research of Luo Kangzhi, and data were provided by Wu Yusheng and Xiang Kebiao in Huanggang village

The income of ducks is not considered here since the number of ducks in each household varies greatly

Straw income: Because the straw of KSR has good flexibility, Dong people often use it as weaving material, such as in brooms and straw sandals, or as dyeing materials after burning

## Discussion

### Traditional agricultural management of KSR in Dong community

KSR, as a kind of rice landraces, plays an important role in rice cultivation in minority areas of China. Male and female labor division is different in the traditional agricultural management of KSR; especially, Dong women have made outstanding contributions to the breeding, inheritance and traditional utilization of KSR, which is the same as the research results of Pu et al*.* [54]. In addition, the traditional methods of multi-planting, traditional storage management and rice–fish–duck coexistence management adopted by Dong people all have profound connotation of ethnoecology. The Dong people have always believed that the agricultural ecosystem formed under this traditional management is wealth (Oux Naemx). We hope to find an effective way to protect and develop the traditional genetic resources of Dong people and carry forward the traditional ethnic ecological culture, so as to provide practical experience and theoretical basis for the management and sustainable use of traditional agriculture in Dong area and even the whole ethnic area; Meanwhile, we hope to promote the recognition and support of the positive role and contribution of traditional agricultural management in maintaining the diversity of germplasm resources, passing on the traditional seed knowledge, protecting biodiversity, ensuring the sustainable development of society and addressing climate change; what’s more, we also hope that the valuable knowledge and ideas discovered in this research can provide reference and guidance for promoting the sustainable development of society, economy, culture and make certain contributions to the construction of ecological civilization today.

### Ethnic minority traditional culture and diversity of agricultural genetic resources

Human civilization began with farming, and farming civilization began with the domestication of seeds. Crop variety diversity is indispensable for maintaining ecosystem stability and resilience and meeting the diverse needs of human society [55]. In thousands of years of agricultural history and civilization, ethnic minorities have preserved and accumulated rich and diverse seeds from generation to generation through continuous seed retention, selection and replacement under their unique traditional cultural background. They also cope with innovation in nature through breeding and cultivation, forming a rich and diverse agricultural ecosystem [56, 57].

The traditional glutinous rice cultivation system of Dong people is a special cultivation mode formed in long-term production practice according to the ecological adaptability and production conditions of glutinous rice. Similarly, Westengen et al*.* [58] used linguistic and anthropological evidence to analyze the role of geographical, ecological, historical and cultural factors in the formation of sorghum (*Sorghum bicolor*) genetic structure, and the results showed that traditional culture played a key role in the formation of sorghum genetic structure in Africa. In addition, traditional ethnic culture plays a positive role in protecting the diversity of agricultural genetic resources. The diversity of landraces is closely related to cultural customs and even to dietary habits or taste preferences of different ethnic groups [59]. Song et al. [60] found that the Yi people in Liangshan, China, chose different areas to plant tartary buckwheat (*Fagopyrum tataricum*) of different varieties to adapt to climate change and changes at different altitudes based on their traditional cultural knowledge, which greatly protected the diversity of local tartary buckwheat (*Fagopyrum tataricum*) landraces. Ma et al. [16] studied the landraces of traditional food crops in typical villages of ecological migration in Ningxia, China, and found that the traditional food plants retained by villagers were closely related to the traditional food culture, and the traditional culture played an important role in promoting the preservation of migrant farm germplasm resources. Feng et al. [61] found that there was a highly significant correlation between the population proportion of the Dai, Hani, Lahu and Bulang ethnic groups in Yunnan and the richness of rice landraces (*P* < 0.01). In this study, the reason why Dong people continue to grow these landraces of KSR is not only because the local climate and environmental conditions are suitable but also, more importantly, because KSR is a very important cultural plant [62]. Thus, from a scientific point of view, traditional management of ethnic minorities still has important ecological significance, scientific value and great reference value for the comprehensive development of agriculture, forestry and animal husbandry.

### The significance of Dong traditional agricultural management to natural resource protection

Natural resources are the most important material basis for human survival and development [63], and human management of natural resources is an immutable theme [5]. Article 10(c) of the Convention on Biological Diversity states “to protect and encourage the use of living resources in accordance with traditional cultural practices and consistent with the requirements of sustainable use.” Therefore, the full protection of these traditional technologies related to the sustainable utilization of biological resources is an important part of biodiversity conservation [4]. The traditional agricultural management of Dong people provides a suitable place for the survival of local biodiversity; for example, terraced fields and fish ponds are kept in the most natural and primitive state as much as possible. Their lives follow certain local ethnic customary laws [64] to ensure that all living things have sustained vitality. This traditional agricultural management skill is the product of Dong people adapting to the unique natural ecological environment in long-term historical development. It is not only the direct embodiment of Dong traditional agriculture but also an important component of Dong traditional culture and distinct national characteristics. In addition, the native ecosystem of the Dong nationality belongs to mainly a subtropical evergreen broad-leaved forest ecosystem, only a few areas are wetland ecosystems, and its ecological structure is relatively simple [53]. However, the Dong people use special farming methods to build terraces on hillsides, creating a unique artificial wetland ecosystem. Then, they took advantage of the different properties of different landraces of KSR and planted them in suitable conditions. (For example, some were insensitive to light, some were tolerant of cold, and some were adapted to fields with poor soil fertility.) In this case, the capacity of paddy fields to withstand natural risks can be enhanced.

The traditional agricultural management of Dong people also plays an important role in water resource management [51]. At present, the uneven spatial distribution of water resources is the primary problem for global water resource management, but Dong people have formed a set of sustainable development review agroecosystems around KSR cultivation and fish and duck breeding. In this system, the traditional agricultural skills of Dong people enable solid, gaseous and liquid water to realize orderly circulation [13]. Therefore, the statute of Dong traditional rice agronomy has played a major role in water conservation and ecological maintenance. This kind of traditional agronomy is not only limited to the production and living of minority nationality areas or indigenous and local communities (ILCs) but also widely used in modern agricultural scientific research and environmental protection, such as scientific research on climate change response [65, 66], community resource management and sustainable utilization [7, 13] and agricultural transformation [52].

## Conclusions

The traditional agricultural management of the KSR of Dong people in southeast Guizhou developed under a specific environment and cultural background. Dong people have cultivated a large number of KSR landraces for thousands of years. In our study, all farmer families retained and planted four to six KSR landraces. In a sense, KSR is the material basis of Dong people's lives and gives birth to the unique ethnic customs and lifestyles of the Dong people. Furthermore, the genetic diversity of KSR and the original ethnic culture in southeast Guizhou promote each other, and the unique natural environment and the rich original ethnic culture in this area also promote the inheritance and protection of KSR. On this basis, Dong people have formed a complete set of traditional agricultural management systems with ecological connotations, and the Dong language expresses it as “Daeml Yav” and “Oux Naemx,” which symbolized wealth in the Dong language. In Dong community, this traditional agricultural management system, with their wealth value, plays a very important role in coping with water shortages and natural disasters and improving farmers' economic benefits, which is the result of Dong people adapting to the local environmental conditions for thousands of years and the needs of the national traditional culture in inheritance and development.

Human beings have experienced the transformation from a fishing, hunting and gathering civilization to a farming civilization and then to an industrial civilization over thousands of years. People who live in ethnic minority areas and indigenous and local communities are often ignored by the public because they are in politically and economically underdeveloped fringe areas [67]. However, these areas are often extremely rich in biodiversity and cultural diversity [68, 69] and are one of the main habitats of wild animals and plants, as well as the main distribution areas of biological genetic resources. The people of different nationalities in these regions have created rich and colorful traditional knowledge in the process of protecting and sustainably using biological resources, which is worthy of reference for modern society. In the long-term research on traditional KSR culture and traditional agricultural management of Dong people, we found that Dong ecological culture is not only an objective existence but also has rich connotation and profound exploration value, which is an extremely precious and valuable national characteristic of the culture. Therefore, we encourage interdisciplinary research in the natural and social sciences, in-depth study of the ecological culture of ethnic areas and excavation of the common treasures that contribute to the development of all humankind.

## Data Availability

All data generated or analyzed during this study are included in this published article.

## References

[1] Li BP, Xue DY (2020). Bio-cultural diversity in ethnic areas: based on the investigation of Bio-culture in Jinxiu, Guangxi, China. Guizhou Soc Sci.

[2] Hill R, Cullen-Unsworth LC, Talbot LD, Mcintyre-Tamwoy S (2011). Empowering Indigenous peoples’ biocultural diversity through World Heritage cultural landscapes: a case study from the Australian humid tropical forests. Int J Herit Stud.

[3] Plieninger T, Kohsaka R, Bieling C, Hashimoto S, Kamiyama C, Kizos T, Penker M, Kieninger P, Shaw BJ, Sioen GB, Yoshida Y, Saito O (2017). Fostering biocultural diversity in landscapes through place-based food networks: a “solution scan” of European and Japanese models. Sustain Sci.

[4] Liu CH, Yang JB, Yin L (2021). Progress, achievements and prospects of biodiversity rotection in Yunnan Province. Biodivers Sci.

[5] Long CL (2009). Traditional management of natural resources in ethnic areas.

[6] Xue DY (2019). Conservation and outlook of traditional knowledge associated with biodiversity. Biodivers Sci.

[7] Liu GD, Tian K, Yuan XZ, Sun JF (2016). Traditional Chinese ecological wisdom and its practical meaning: a case study of the river system in Lijiang Old Town. Acta Ecol Sin.

[8] Ding LB, Ma N, Wang GP, He SY, Min QW (2019). Visual analysis of hotspots and emerging trends in traditional knowledge associated with biodiversity. Biodivers Sci.

[9] Shrestha PM, Dhillion SS (2006). Diversity and traditional knowledge concerning wild food species in a locally managed forest in Nepal. Agrofor Syst.

[10] Parrotta JA, Agnoletti M (2007). Traditional forest knowledge: challenges and opportunities. For Ecol Manag.

[11] Wu JY, Xue DY (2017). Important issues concerning the national legislation of access to genetic resources and benefit-sharing. Biodivers Sci.

[12] Camacho LD, Gevaña DT, Carandang AP, Camacho SC (2016). Indigenous knowledge and practices for the sustainable management of Ifugao forests in Cordillera, Philippines. Int J Biodivers Sci Ecosyst Serv Manag.

[13] Yang JB, Xia JX, Feng JC, Guo L, Shi S, Xue DY (2018). Water resource management in the Hani Rice Terraces agro-ecosystem from an ethnoecological perspective. Acta Ecol Sin.

[14] Pradhan A, Ormsby AA (2020). Biocultural conservation in the sacred forests of Odisha, India. Environ Conserv.

[15] Shao H, Hill R, Xue DY, Yang JB (2021). In situ conservation of traditional vegetable diversity in Wa homegardens in southwestern Yunnan, China. J Ethnobiol Ethnomed.

[16] Ma Y, Luo BS, Zhu Q, Ma DX, Wen Q, Feng JC, Xue DY (2019). Changes in traditional ecological knowledge of forage plants in immigrant villages of Ningxia, China. J Ethnobiol Ethnomed.

[17] Franco FM (2015). Calendars and ecosystem management: some observations. Hum Ecol.

[18] Pieroni A, Soukand R (2019). Ethnic and religious affiliations affect traditional wild plant foraging in Central Azerbaijan. Genet Resour Crop Evol.

[19] Wang YJ, Wang YL, Jiao AX, Caiji ZM, Yang JB, Ruan RC, Xue DY (2015). Influence of national traditional culture on crop genetic diversity——take an example of Kam Sweet Rice in Liping county of Guizhou province. J Nat Resour.

[20] Allen T, Prosperi P, Cogill B, Flichman G (2014). Agricultural biodiversity, social ecological systems and sustainable diets. Proc Nutr Soc.

[21] Pilbeam V, van Kerkhoff L, Weir T (2019). Conservation decision-making in Palau: an example of the parallel working of scientific and traditional ecological knowledge. Environ Manag.

[22] Villa TCC, Maxted N, Scholten M (2005). Defining and identifying crop landraces. Plant Gent Resour-C.

[23] Pusadee T, Jamjod S, Chiang YC, Rerkasem B, Schaal BA (2009). Genetic structure and isolation by distance in a landrace of Thai rice. PNAS.

[24] Cui D, Li JM, Tang CF, Ma X, Yu TQ, Ma XD, Zhang EL, Cao GL, Xu FR, Qiao YL, Dai LY, Han LZ (2016). Diachronic analysis of genetic diversity in rice landraces under on-farm conservation in Yunnan, China. Theor Appl Genet.

[25] Zong Y, Chen Z, Innes JB, Chen C, Wang Z, Wang H (2007). Fire and flood management of coastal swamp enabled first rice paddy cultivation in east China. Nature.

[26] Yamanaka S, Nakamura I, Watanabe KN, Sato Y (2004). Identification of SNPs in the waxy gene among glutinous rice cultivars and their evolutionary significance during the domestication process of rice. Theor Appl Genet.

[27] Ruan RC, Chen HC, You JM, Zhu YQ, Zhu M, Chen NG (2007). Current status and prospects of conservation for rice genetic resources in Guizhou. Seeds.

[28] Ruan RC, Chen HC, Chen NG, You JM, Chen F, Jiao AX (2015). Progress on the research and utilization of rice germplasm resources in Guizhou in the 21st century. J Mt Agric Biol.

[29] Khan A, Goldringer I, Thomas M (2020). Management practices and breeding history of varieties strongly determine the fine genetic structure of crop populations: a case study based on European wheat populations. Sustainability (Basel, Switzerland).

[30] Singh R, Singh G (2017). Traditional agriculture: a climate-smart approach for sustainable food production. Energy Ecol Environ.

[31] Abate T, van Huis A, Ampofo JKO (2000). Pest management strategies in traditional agriculture: an African perspective. Annu Rev Entomol.

[32] Jarvis D (2016). Crop genetic diversity in the field and on the farm: principles and applications in research practices.

[33] Aesomnuk W, Ruengphayak S, Ruanjaichon V, Sreewongchai T, Malumpong C, Vanavichit A, Arikit S (2021). Estimation of the genetic diversity and population structure of Thailand’s rice landraces using SNP markers. Agronomy (Basel).

[34] Song YJ, Fang Q, Jarvis D, Bai KY, Liu DM, Feng JC, Long CL (2019). Network analysis of seed flow, a traditional method for conserving tartary buckwheat (*Fagopyrum tataricum*) landraces in Liangshan, Southwest China. Sustainability (Basel, Switzerland).

[35] Cherfas J, Hodgkin T, Frison E (2011). Agricultural biodiversity is essential for a sustainable improvement in food and nutrition security. Sustainability (Basel, Switzerland).

[36] Srivastava P, Singh R, Tripathi S, Raghubanshi AS (2016). An urgent need for sustainable thinking in agriculture: an Indian scenario. Ecol Ind.

[37] You XL (2010). Cultural history of rice cultivation in China.

[38] Liu ZF (2014). An introduction to rice culture in China.

[39] Lei QY, Zhou JJ, Xiong Y, Zhang WH, Luo J, Long CL (2021). Genetic diversity evaluation and conservation of Kam Fragrant Glutinous Rice (*Oryza sativa* L.) germplasm in Southeast Guizhou, China. Plants (Basel, Switzerland).

[40] Wang YJ, Jiao AX, Chen HC, Ma XD, Cui D, Han B, Ruan RC, Xue DY, Han LZ (2018). Status and factors influencing on-farm conservation of Kam Sweet Rice (*Oryza sativa* L.) genetic resources in southeast Guizhou Province, China. J Ethnobiol Ethnomed.

[41] Zhou JB, Wu SY, Chen M, Lei QY (2019). Evolution and differentiation of Kam Sweet Rice. China Rice.

[42] Lei QY, Bai HF, Zhang WH, Zhou JJ (2009). Study on protection of the diversity of prototypical ethnic culture and the genetic diversity of glutinous rice resources in Southeast of Guizhou. J Anhui Agric Sci.

[43] Lei QY, Zhang WH, Sun J, Yang MX, Zhou JJ (2013). Traditional management and utilization of glutinous rice genetic resources in Southeast Guizhou. Plant Divers Resour.

[44] Richard S (2008). Intellectual property: Chinese province crafts pioneering law to thwart biopiracy. Science.

[45] Zhou JB, Yang T, Lei QY, Yan HG (2020). Kam sweet rice in Miao and Dong folk culture in Qiandongnan prefecture. J Xingyi Norm Univers for Nationali.

[46] Compiled by National Bureau of Statistics of China. China Statistical Yearbook-2021.2021. http://www.stats.gov.cn/tjsj/ndsj/2021/indexch.htm. Accessed 2 Dec 2021.

[47] Wang YH, Wang C (2017). Common research methods in ethnobotany.

[48] Zheng DS, Liu X, Lu XX (2007). Technical regulations of crop germplasm resources.

[49] International Society of Ethnobiology 2006. International Society of Ethnobiology Code of Ethics (with 2008 additions). http://ethnobiology.net/code-of-ethics/. Accessed 25 Feb 2022.

[50] Tardío J, Pardo-de-Santayana M (2008). Cultural importance indices: a comparative analysis based on the useful wild plants of Southern Cantabria (Northern Spain). Econ Bot.

[51] Chen XL, Deng MW (2013). Research on ecological culture of Dong nationality in China.

[52] Huang WJ, Gilbert S, Poulev A, Acosta K, Lebeis S, Long CL, Lam E (2020). Host-specific and tissue-dependent orchestration of microbiome community structure in traditional rice paddy ecosystems. Plant Soil.

[53] Luo KZ (2014). Preservation and innovation: adapting tradition to modern in Huanggang Dong Village of Liping County.

[54] Pu K, Long XY (2012). Dong women and KSR resources Inheritance and protection: a case study of Liping, Guizhou. J Southwest Univ for Nationalities (Humanit Soc Sci).

[55] Bardsley D (2003). Risk alleviation via in situ agrobiodiversity conservation: drawing from experiences in Switzerland, Turkey and Nepal. Agric Ecosyst Environ.

[56] Long CL, Zhou YL (2001). Indigenous community forest management of Jinuo people’s swidden agroecosystems in southwest China. Biodivers Conserv.

[57] Pluckneet DL, Smith NJH, Williams JT, Anishetty NM (1983). Crop germplasm conservation and developing countries. Science.

[58] Westengen OT, Okongoc MA, Onekd L, Berge T, Upadhyaya H, Birkeland B, Khalsa SDK, Ringa KH, Stenseth NC, Brysting AK (2014). Ethnolinguistic structuring of sorghum genetic diversity in Africa and the role of local seed systems. PNAS.

[59] Bellon MR (2004). Conceptualizing interventions to support on-farm genetic resource conservation. World Dev.

[60] Song YJ, Dong YM, Wang J, Feng JC, Long CL (2019). Tartary buckwheat (*Fagopyrum tataricum Gaertn*.) landraces cultivated by Yi people in Liangshan, China. Genet Resour Crop Evol.

[61] Feng JM, He HM, Zhu YY, Li CY (2010). Correlation between geographic patterns of cultivar diversity of rice (*Oryza sativa* L) and environmental factors, local culture in Yunnan. J Yunnan Agric Univ.

[62] Heinrich M, Ankli A, Frei B, Weimann C, Sticher O (1998). Medicinal plant in Mexico: healers’ consensus and cultural important. Soc Sci Med.

[63] Venables AJ (2016). Using natural resources for development: why has it proven so difficult?. J Econ Perspect.

[64] Xue DY, Guo L (2009). On concepts and protection of traditional knowledge. Biodivers Sci.

[65] Liu CH, Cheng G, Xue DY, Chen XL (2013). An overview on traditional knowledge with the linkage of climate change. J Yunnan Agric Univers (Nat Sci).

[66] Yin L (2011). Cognition and response of Tibetan to climate change-an investigation of Guonian Village, Deqin County, Yunnan Province. Ideol Front.

[67] Salick J, Byg A (2007). Indigenous peoples and climate Change.

[68] Long CL, Pei SJ (2003). Cultural diversity promotes conservation and application of biological diversity. Acta Bot Yunnanica.

[69] Pei SJ (2011). Traditional culture and biodiversity conservation. Bull Chin Acad Sci.

